# Supplementary material for: Past conservation efforts reveal which actions lead to positive outcomes for species
Source: PLoS Biol. 2025 Mar 18;23(3):e3003051. doi: 10.1371/journal.pbio.3003051 (PMC12135918; doi:10.1371/journal.pbio.3003051)
Supplement: S1 Text — (DOCX) [file pbio.3003051.s001.docx]

Freshwater fish consisted of species of Actinopterygii coded with system ‘freshwater’. Selected crustacea consisted species of freshwater crabs, shrimps and crayfishes and lobsters. Selected gastropods consisted species of abalones and cone snails. Selected marine fish consisted of species of Clupeiformes, blennies, boarfishes, butterflyfishes, croakers and drums, Stomiiformes, emperor breams, filefishes, groupers, grunts, Saccopharyngiformes, Carangidae, lanternfishes, Aulopiformes, louvar, Tetraodontidae, seabreams, porgies and picarels, snappers, sturgeons and paddlefishes, surgeonfishes, tangs and unicorn fishes, syngathiform fishes, seamoths, tarpons and ladyfishes, tunas, billfishes and swordfish, wrasses and parrotfishes. Warm-water reef-building corals consisted of species in the Class Hydrozoa and Family Milleporidae, or Families: Acroporidae,, Agariciidae, Astrangiidae, Astrocoeniidae, Cladocoridae, Coscinaraeidae, Diploastraeidae, Euphylliidae, Faviidae, Fungiidae, Helioporidae, Leptastreidae, Lobophylliidae, Meandrinidae, Merulinidae, Montastraeidae, Oculinidae, Oulastreidae, Plerogyridae, Plesiastreidae, Pocilloporidae, Poritidae, Psammocoridae, Rhizangiidae, Tubiporidae or Turbinoliidae; or Genus: Heterocyathus, Balanophyllia, Duncanopsammia, Heterosammia, Turbinaria, Pachyseris or Solenastrea.
