# Supplementary material for: Past conservation efforts reveal which actions lead to positive outcomes for species
Source: PLoS Biol. 2025 Mar 18;23(3):e3003051. doi: 10.1371/journal.pbio.3003051 (PMC12135918; doi:10.1371/journal.pbio.3003051)
Supplement: S2 Text — (DOCX) [file pbio.3003051.s002.docx]

Threat data were aggregated up to level 1 of the IUCN threats classification system [1], with the exception of Biological resource use, for which logging and wood harvesting and gathering terrestrial plants, were separated from hunting/collecting and fishing/harvesting aquatic resources, given the substantial impacts of logging and harvesting of plants on terrestrial animal species’ habitats. Future threats, or those with negligible impact, were excluded using the timing and severity coding in the IUCN threat information (threats without timing or severity coding were retained to avoid excluding important threats). To reduce the number of variables for use in modelling, threats at level 1 (except Biological resource use) were grouped into the following higher level variables: *habitat loss or degradation* (Residential and commercial development, Agriculture and aquaculture, Energy production and mining, transport and service corridors, Biological resource use: logging and wood harvesting and gathering terrestrial plants, Human intrusions and disturbance, Natural system modification and Geological Events), *hunting or fishing* (other categories of Biological resource use), *invasive or problematic species and diseases* (Invasive & problematic native species, genes and diseases), *pollution* (Pollution) and *climate change* (Climate change and severe weather).

References

1. Salafsky N, Salzer D, Stattersfield AJ, Hilton-Taylor C, Neugarten R, Butchart SHM, et al. A Standard Lexicon for Biodiversity Conservation: Unified Classifications of Threats and Actions. Conservation Biology. 2008;22: 897–911. doi:10.1111/j.1523-1739.2008.00937.x
