# Supplementary material for: Past conservation efforts reveal which actions lead to positive outcomes for species
Source: PLoS Biol. 2025 Mar 18;23(3):e3003051. doi: 10.1371/journal.pbio.3003051 (PMC12135918; doi:10.1371/journal.pbio.3003051)
Supplement: S3 Text — (DOCX) [file pbio.3003051.s003.docx]

To simplify the number of variables for analysis, where relating to the same action, the conservation actions in place were recoded to align broadly with level 1 of the IUCN Actions classification scheme. The actions in place were recoded as follows: *In protected area* (presence in at least one protected area, any threshold of protection > 0), *Area management plan* (an area-based regional management plan, typically relating to site management e.g. of a protected area), *Control of invasive or problematic species* (invasive species control/prevention), *Species management* (species action/recovery plan, species harvest management plan), *Reintroduction or translocation* (reintroduction/introduction/translocation), *Awareness and education* (recent education/awareness programmes), International l*egislation or trade control* (included in international legislation, subject to international management or trade controls), *Captive breeding* or *Monitoring* (included in monitoring schemes). Note the action *Inside conservation areas* (which is intended to reflect whether sites of biodiversity importance have been identified for the species) was excluded as expert assessors’ information suggests that it has been applied inconsistently between groups, thus including uncertainty in the accuracy of this dataset.
