# Supplementary material for: Past conservation efforts reveal which actions lead to positive outcomes for species
Source: PLoS Biol. 2025 Mar 18;23(3):e3003051. doi: 10.1371/journal.pbio.3003051 (PMC12135918; doi:10.1371/journal.pbio.3003051)
Supplement: S4 Text — (DOCX) [file pbio.3003051.s004.docx]

Since 1980, 288 species are reported to have undergone genuine improvements in their IUCN Red List category through conservation or other reasons (i.e. to have qualified for downlisting), consisting of 122 amphibians (since 1980), 111 birds (since 1988), 32 mammals (since 1996), 2 warm-water reef-forming corals (since 1996), and 6 reptiles, 6 marine fish, 4 freshwater fish, 3 cartilaginous fish and 2 snails (all since 2016). Of the 13 species without coded conservation actions, 11 had actions indicated in the narrative text, leaving only two species with no apparent actions in place, *Craugastor taurus* (amphibian) and *Notomys cervinus* (mammal).

Improvement in Red List category was attributed to conservation action in 91 species (71.7% of 127 species of mammal and birds), comprising of 75 bird and 16 mammal species. Only seven bird species, such as Woodford’s rail, *Hypotaenidia woodfordi*, and Mewing kingfisher, *Todiramphus ruficollaris*, had only non-conservation reasons for improvement, relating to natural recovery following severe weather events, land abandonment or cessation of other activities. For the remaining 24 bird and 5 mammal species, the reason for improvement was not clearly attributed.
