# Supplementary material for: Past conservation efforts reveal which actions lead to positive outcomes for species
Source: PLoS Biol. 2025 Mar 18;23(3):e3003051. doi: 10.1371/journal.pbio.3003051 (PMC12135918; doi:10.1371/journal.pbio.3003051)
Supplement: S1 Table — For analysis of actions across IUCN Red List categories within taxa, this could not be evaluated for Merostomata, Myxini, Sarcopterygii and warm-water reef building corals. (DOCX) [file pbio.3003051.s005.docx]

| **Taxon** | **IUCN Red List category** | **Residuals** | **p value** | **More/less likely** |
| --- | --- | --- | --- | --- |
| Aves | all | 60.56487 | <0.001*** | > |
| Amphibia | all | 50.95389 | <0.001*** | > |
| Reptilia | all | 41.35287 | <0.001*** | > |
| Warm-water reef-building corals | all | 27.55593 | <0.001*** | > |
| Merostomata | all | -0.07375 | 1 | NS |
| Sarcopterygii | all | -0.10431 | 1 | NS |
| Mammalia | all | -2.35021 | 0.600 | NS |
| Petromyzonti | all | -4.12444 | 0.001** | < |
| Myxini | all | -7.49457 | <0.001*** | < |
| Chondrichthyes | all | -10.5467 | <0.001*** | < |
| Selected marine fish | all | -16.4605 | <0.001*** | < |
| Selected gastropods | all | -22.7301 | <0.001*** | < |
| Cephalopoda | all | -26.4433 | <0.001*** | < |
| Dragonflies & damselflies | all | -28.0223 | <0.001*** | < |
| Selected crustacea | all | -42.086 | <0.001** | < |
| Freshwater fish | all | -69.1586 | <0.001*** | < |
| All | Near Threatened | 10.63879 | <0.001*** | > |
|  | Vulnerable | 0.950171 | 1 | NS |
|  | Endangered | -2.27432 | 0.184 | NS |
|  | Critically Endangered | -10.2608 | <0.001*** | < |
| Amphibia | Near Threatened | 6.241307 | <0.001*** | > |
|  | Vulnerable | 6.912726 | <0.001*** | > |
|  | Endangered | -1.36612 | 1 | NS |
|  | Critically Endangered | -10.3376 | <0.001*** | < |
| Aves | Near Threatened | -3.98213 | <0.001*** | < |
|  | Vulnerable | 2.82727 | 0.038* | > |
|  | Endangered | 0.971679 | 1 | NS |
|  | Critically Endangered | 0.919876 | 1 | NS |
| Cephalopoda | Near Threatened | 1.707825 | 0.701 | NS |
|  | Vulnerable | -0.68313 | 1 | NS |
|  | Endangered | -0.68313 | 1 | NS |
|  | Critically Endangered | -0.44096 | 1 | NS |
| Chondrichthyes | Near Threatened | -2.39078 | 0.134 | NS |
|  | Vulnerable | 0.188703 | 1 | NS |
|  | Endangered | 0.452642 | 1 | NS |
|  | Critically Endangered | 1.960376 | 0.399615 | NS |
| Dragonflies & damselflies | Near Threatened | 3.444471 | 0.005** | > |
|  | Vulnerable | 1.079743 | 1 | NS |
|  | Endangered | -2.28617 | 0.178 | NS |
|  | Critically Endangered | -3.03665 | 0.019* | < |
| Freshwater fish | Near Threatened | 5.923018 | <0.001*** | > |
|  | Vulnerable | -2.7349 | 0.050* | < |
|  | Endangered | 1.777481 | 0.604 | NS |
|  | Critically Endangered | -4.63233 | <0.001*** | < |
| Mammalia | Near Threatened | 0.530321 | 1 | NS |
|  | Vulnerable | 1.365813 | 1 | NS |
|  | Endangered | -2.34108 | 0.154 | NS |
|  | Critically Endangered | 0.681128 | 1 | NS |
| Petromyzonti | Near Threatened | -1.41421 | 1 | NS |
|  | Vulnerable | -1.41421 | 1 | NS |
|  | Endangered | 2.165064 | 0.243 | NS |
|  | Critically Endangered | 0.547723 | 1 | NS |
| Reptilia | Near Threatened | 3.939116 | <0.001*** | > |
|  | Vulnerable | 2.242336 | 0.200 | NS |
|  | Endangered | 0.60195 | 1 | NS |
|  | Critically Endangered | -7.64468 | <0.001*** | < |
| Selected crustacea | Near Threatened | -1.21407 | 1 | NS |
|  | Vulnerable | -2.62624 | 0.069 | NS |
|  | Endangered | 1.080334 | 1 | NS |
|  | Critically Endangered | 3.019929 | 0.020* | > |
| Selected gastropods | Near Threatened | -1.86877 | 0.493 | NS |
|  | Vulnerable | -0.29053 | 1 | NS |
|  | Endangered | -0.50381 | 1 | NS |
|  | Critically Endangered | 3.854612 | <0.001*** | > |
| Selected marine fish | Near Threatened | 0.487054 | 1 | NS |
|  | Vulnerable | -1.65371 | 0.785 | NS |
|  | Endangered | 0.305829 | 1 | NS |
|  | Critically Endangered | 1.613296 | 0.853 | NS |
