# Supplementary material for: Past conservation efforts reveal which actions lead to positive outcomes for species
Source: PLoS Biol. 2025 Mar 18;23(3):e3003051. doi: 10.1371/journal.pbio.3003051 (PMC12135918; doi:10.1371/journal.pbio.3003051)
Supplement: S2 Table — If actions are absent, they were not reported for any species in that group. (DOCX) [file pbio.3003051.s006.docx]

| **Taxon** | **IUCN Red List category** | **Action type** | **Residuals** | **p value** | **More/less likely** |
| --- | --- | --- | --- | --- | --- |
| All | All | In protected area | 475.64 | 0.000 | > |
|  |  | Legislation/Trade control | 2.25 | 0.491 | NS |
|  |  | Monitoring scheme | -45.33 | 0.000 | < |
|  |  | Species management plan | -52.71 | 0.000 | < |
|  |  | Awareness | -59.63 | 0.000 | < |
|  |  | Captive breeding | -60.33 | 0.000 | < |
|  |  | Area management plan | -61.53 | 0.000 | < |
|  |  | Harvest management plan | -62.99 | 0.000 | < |
|  |  | Invasive species control | -66.04 | 0.000 | < |
|  |  | (Re)introduced/translocated | -69.33 | 0.000 | < |
| Amphibia | All | In protected area | 232.35 | 0.000 | > |
|  |  | Legislation/Trade control | -19.91 | 0.000 | < |
|  |  | Captive breeding | -22.91 | 0.000 | < |
|  |  | Invasive species control | -24.94 | 0.000 | < |
|  |  | Awareness | -25.61 | 0.000 | < |
|  |  | Monitoring scheme | -26.38 | 0.000 | < |
|  |  | Species management plan | -26.67 | 0.000 | < |
|  |  | Area management plan | -28.19 | 0.000 | < |
|  |  | (Re)introduced/translocated | -28.40 | 0.000 | < |
|  |  | Harvest management plan | -29.33 | 0.000 | < |
| Aves | All | In protected area | 205.24 | 0.000 | > |
|  |  | Legislation/Trade control | 34.71 | 0.000 | > |
|  |  | Monitoring scheme | -2.86 | 0.083 | < |
|  |  | Species management plan | -16.76 | 0.000 | < |
|  |  | Awareness | -31.61 | 0.000 | < |
|  |  | Invasive species control | -33.68 | 0.000 | < |
|  |  | Captive breeding | -34.31 | 0.000 | < |
|  |  | (Re)introduced/translocated | -38.90 | 0.000 | < |
|  |  | Area management plan | -40.35 | 0.000 | < |
|  |  | Harvest management plan | -41.48 | 0.000 | < |
| Cephlapoda | All | In protected area | 5.94 | 0.000 | > |
|  |  | Legislation/Trade control | -1.82 | 0.554 | NS |
|  |  | Monitoring scheme | -1.82 | 0.554 | NS |
|  |  | Harvest management plan | -2.30 | 0.171 | NS |
| Chondrichthyes | All | In protected area | 36.45 | 0.000 | > |
|  |  | Harvest management plan | 6.15 | 0.000 | > |
|  |  | Legislation/Trade control | -3.64 | 0.004 | < |
|  |  | Monitoring scheme | -4.16 | 0.001 | < |
|  |  | Species management plan | -7.64 | 0.000 | < |
|  |  | Area management plan | -7.90 | 0.000 | < |
|  |  | Awareness | -8.54 | 0.000 | < |
|  |  | Captive breeding | -10.73 | 0.000 | < |
| Dragonflies & damselflies | All | In protected area | 128.79 | 0.000 | > |
|  |  | Species management plan | -17.86 | 0.000 | < |
|  |  | Monitoring scheme | -18.00 | 0.000 | < |
|  |  | Area management plan | -18.34 | 0.000 | < |
|  |  | Awareness | -18.34 | 0.000 | < |
|  |  | Legislation/Trade control | -18.61 | 0.000 | < |
|  |  | Invasive species control | -18.68 | 0.000 | < |
|  |  | Harvest management plan | -18.96 | 0.000 | < |
| Freshwater fish | All | In protected area | 156.47 | 0.000 | > |
|  |  | Area management plan | -4.24 | 0.000 | < |
|  |  | Species management plan | -15.95 | 0.000 | < |
|  |  | Captive breeding | -16.84 | 0.000 | < |
|  |  | Harvest management plan | -17.74 | 0.000 | < |
|  |  | Awareness | -18.94 | 0.000 | < |
|  |  | Monitoring scheme | -19.63 | 0.000 | < |
|  |  | (Re)introduced/translocated | -20.41 | 0.000 | < |
|  |  | Invasive species control | -20.99 | 0.000 | < |
|  |  | Legislation/Trade control | -21.73 | 0.000 | < |
| Mammalia | All | In protected area | 113.56 | 0.000 | > |
|  |  | Legislation/Trade control | 25.91 | 0.000 | > |
|  |  | Captive breeding | -12.21 | 0.000 | < |
|  |  | Awareness | -12.84 | 0.000 | < |
|  |  | Monitoring scheme | -15.40 | 0.000 | < |
|  |  | Species management plan | -17.13 | 0.000 | < |
|  |  | (Re)introduced/translocated | -18.91 | 0.000 | < |
|  |  | Harvest management plan | -19.70 | 0.000 | < |
|  |  | Area management plan | -20.80 | 0.000 | < |
|  |  | Invasive species control | -22.47 | 0.000 | < |
| Merostomata | All | Awareness | 1.29 | 1.000 | NS |
|  |  | (Re)introduced/translocated | -0.32 | 1.000 | NS |
|  |  | Harvest management plan | -0.32 | 1.000 | NS |
|  |  | In protected area | -0.32 | 1.000 | NS |
|  |  | Monitoring scheme | -0.32 | 1.000 | NS |
| Petromyzonti | All | In protected area | 1.69 | 0.910 | NS |
|  |  | Awareness | -0.42 | 1.000 | NS |
|  |  | Legislation/Trade control | -0.42 | 1.000 | NS |
|  |  | Monitoring scheme | -0.42 | 1.000 | NS |
|  |  | Species management plan | -0.42 | 1.000 | NS |
| Reptilia | All | In protected area | 236.57 | 0.000 | > |
|  |  | Legislation/Trade control | -1.85 | 1.000 | NS |
|  |  | Area management plan | -28.28 | 0.000 | < |
|  |  | Species management plan | -28.59 | 0.000 | < |
|  |  | Captive breeding | -28.70 | 0.000 | < |
|  |  | Awareness | -28.74 | 0.000 | < |
|  |  | Invasive species control | -29.09 | 0.000 | < |
|  |  | Monitoring scheme | -29.44 | 0.000 | < |
|  |  | (Re)introduced/translocated | -30.40 | 0.000 | < |
|  |  | Harvest management plan | -31.48 | 0.000 | < |
| Sarcopterygii | All | Legislation/Trade control | 1.66 | 0.586 | NS |
|  |  | In protected area | -0.83 | 1.000 | NS |
|  |  | Monitoring scheme | -0.83 | 1.000 | NS |
| Selected crustacea | All | In protected area | 45.53 | 0.000 | > |
|  |  | Harvest management plan | 4.87 | 2.3e-05 | > |
|  |  | Area management plan | -3.30 | 0.020 | < |
|  |  | Monitoring scheme | -4.55 | 0.000 | < |
|  |  | Awareness | -6.28 | 0.000 | < |
|  |  | Species management plan | -6.28 | 0.000 | < |
|  |  | (Re)introduced/translocated | -7.06 | 0.000 | < |
|  |  | Legislation/Trade control | -7.06 | 0.000 | < |
|  |  | Captive breeding | -7.85 | 0.000 | < |
|  |  | Invasive species control | -8.01 | 0.000 | < |
| Selected gastropods | All | In protected area | 14.49 | 0.000 | > |
|  |  | Harvest management plan | -1.04 | 1.000 | NS |
|  |  | Species management plan | -1.38 | 1.000 | NS |
|  |  | Monitoring scheme | -1.73 | 1.000 | NS |
|  |  | (Re)introduced/translocated | -2.07 | 0.615 | NS |
|  |  | Area management plan | -2.07 | 0.615 | NS |
|  |  | Captive breeding | -2.76 | 0.092 | < |
|  |  | Awareness | -3.45 | 0.009 | < |
| Selected marine fish | All | In protected area | 115.89 | 0.000 | > |
|  |  | Harvest management plan | 0.59 | 1.000 | NS |
|  |  | Area management plan | -9.01 | 0.000 | < |
|  |  | Legislation/Trade control | -12.19 | 0.000 | < |
|  |  | Awareness | -13.32 | 0.000 | < |
|  |  | Monitoring scheme | -15.16 | 0.000 | < |
|  |  | Species management plan | -15.73 | 0.000 | < |
|  |  | Captive breeding | -15.87 | 0.000 | < |
|  |  | Invasive species control | -17.56 | 0.000 | < |
|  |  | (Re)introduced/translocated | -17.63 | 0.000 | < |
| Warm-water reef building corals | All | In protected area | 63.91 | 0.000 | > |
|  |  | Legislation/Trade control | -15.90 | 0.000 | < |
|  |  | Captive breeding | -16.00 | 0.000 | < |
|  |  | Monitoring scheme | -16.00 | 0.000 | < |
|  |  | Species management plan | -16.00 | 0.000 | < |
| All | Near Threatened | In protected area | 12.79 | 0.000 | > |
|  |  | Area management plan | -0.64 | 1.000 | NS |
|  |  | Legislation/Trade control | -1.18 | 1.000 | NS |
|  |  | Harvest management plan | -1.19 | 1.000 | NS |
|  |  | Monitoring scheme | -2.05 | 1.000 | NS |
|  |  | (Re)introduced/translocated | -2.86 | 0.171 | NS |
|  |  | Invasive species control | -3.04 | 0.096 | NS |
|  |  | Species management plan | -5.31 | 4e-06 | < |
|  |  | Awareness | -5.75 | 0.000 | < |
|  |  | Captive breeding | -8.46 | 0.000 | < |
|  | Vulnerable | In protected area | 2.62 | 0.350 | NS |
|  |  | Legislation/Trade control | 1.07 | 1.000 | NS |
|  |  | Harvest management plan | 0.54 | 1.000 | NS |
|  |  | (Re)introduced/translocated | 0.08 | 1.000 | NS |
|  |  | Species management plan | -0.06 | 1.000 | NS |
|  |  | Invasive species control | -0.64 | 1.000 | NS |
|  |  | Monitoring scheme | -0.72 | 1.000 | NS |
|  |  | Area management plan | -1.24 | 1.000 | NS |
|  |  | Captive breeding | -2.48 | 0.519 | NS |
|  |  | Awareness | -3.43 | 0.024 | < |
|  | Endangered | Awareness | 1.83 | 1.000 | NS |
|  |  | Captive breeding | 1.52 | 1.000 | NS |
|  |  | Invasive species control | 0.38 | 1.000 | NS |
|  |  | (Re)introduced/translocated | 0.04 | 1.000 | NS |
|  |  | In protected area | -0.06 | 1.000 | NS |
|  |  | Area management plan | -0.09 | 1.000 | NS |
|  |  | Legislation/Trade control | -0.13 | 1.000 | NS |
|  |  | Species management plan | -0.61 | 1.000 | NS |
|  |  | Monitoring scheme | -1.46 | 1.000 | NS |
|  |  | Harvest management plan | -1.72 | 1.000 | NS |
|  | Critically Endangered | Captive breeding | 10.42 | 0.000 | > |
|  |  | Awareness | 8.23 | 0.000 | > |
|  |  | Species management plan | 6.58 | 0.000 | > |
|  |  | Monitoring scheme | 4.82 | 5.9e-05 | > |
|  |  | Invasive species control | 3.62 | 0.012 | > |
|  |  | (Re)introduced/translocated | 2.97 | 0.120 | NS |
|  |  | Harvest management plan | 2.68 | 0.297 | NS |
|  |  | Area management plan | 2.29 | 0.878 | NS |
|  |  | Legislation/Trade control | 0.14 | 1.000 | NS |
|  |  | In protected area | -17.02 | 0.000 | < |
