# Supplementary material for: Past conservation efforts reveal which actions lead to positive outcomes for species
Source: PLoS Biol. 2025 Mar 18;23(3):e3003051. doi: 10.1371/journal.pbio.3003051 (PMC12135918; doi:10.1371/journal.pbio.3003051)
Supplement: S3 Table — Positive estimates indicate the variable correlates with a more favorable state, with positively and negatively correlated variables in green and orange font, respectively. The below shows overall model results, models just looking at only species traits, and models for particular taxa with sufficient species and actions to evaluate independently. (DOCX) [file pbio.3003051.s007.docx]

| **Indicator** | **Taxonomic Group(s)** | **Variable type** | **Variable** | **Estimate** | **SE** | **z value** | **p value** |
| --- | --- | --- | --- | --- | --- | --- | --- |
| Global population trend | All comprehensively assessed taxa, excluding Least Concern Species | Traits | log(range) | -0.306 | 0.014 | -21.813 | < 0.001*** |
|  |  |  | IUCN Red List category (linear) | -2.383 | 0.102 | -23.366 | < 0.001*** |
|  |  |  | IUCN Red List category (quadratic) | -0.052 | 0.082 | -0.641 | 0.522 NS |
|  |  |  | IUCN Red List category (cubic) | 0.376 | 0.075 | 5.037 | < 0.001*** |
|  |  |  | Marine (vs terrestrial) | 0.626 | 0.455 | 1.377 | 0.169 NS |
|  |  |  | Terrestrial and marine (vs terrestrial only) | 1.407 | 0.189 | 7.427 | < 0.001*** |
|  |  | Threats | Climate | 0.588 | 0.097 | 6.047 | < 0.001*** |
|  |  |  | Pollution | -0.597 | 0.103 | -5.791 | < 0.001*** |
|  |  |  | Hunting or fishing | -0.659 | 0.103 | -6.392 | < 0.001*** |
|  |  |  | Habitat loss or degradation | -1.215 | 0.092 | -13.268 | < 0.001*** |
|  |  | Actions | Reintroduction or translocation | 1.183 | 0.175 | 6.744 | < 0.001*** |
|  |  |  | Control invasive or problematic species or disease | 0.984 | 0.141 | 6.984 | < 0.001*** |
|  |  |  | Species management plan | 0.847 | 0.130 | 6.518 | < 0.001*** |
|  |  |  | Awareness and education | 0.729 | 0.141 | 5.167 | < 0.001*** |
|  |  |  | Legislation or trade control | 0.620 | 0.112 | 5.531 | < 0.001*** |
|  | All comprehensively assessed taxa, including Least Concern species and only considering species traits | Traits | log(generation length) | -0.084 | 0.037 | -2.271 | 0.023  * |
|  |  |  | log(range) | -0.051 | 0.008 | -6.582 | <0.001  *** |
|  |  |  | IUCN Red List category (linear) | -2.385 | 0.111 | -21.493 | <0.001  *** |
|  |  |  | IUCN Red List category (quadratic) | 1.296 | 0.097 | 13.364 | <0.001  *** |
|  |  |  | IUCN Red List category (cubic) | -0.533 | 0.105 | -5.05 | <0.001  *** |
|  |  |  | IUCN Red List category (^4) | 0.612 | 0.093 | 6.578 | <0.001  *** |
|  |  |  | Marine (vs terrestrial) | 0.333 | 0.354 | 0.942 | 0.346  NS |
|  |  |  | Terrestrial and marine (vs terrestrial only) | 0.799 | 0.088 | 9.109 | <0.001  *** |
|  | Birds | Traits | log(generation length) | 1.120 | 0.175 | 6.393 | <0.001  *** |
|  |  |  | log(range) | -0.272 | 0.025 | -10.791 | <0.001  *** |
|  |  |  | IUCN Red List category (linear) | -1.067 | 0.210 | -5.079 | <0.001  *** |
|  |  |  | IUCN Red List category (quadratic) | 0.083 | 0.179 | 0.462 | 0.644  NS |
|  |  |  | IUCN Red List category (cubic) | 0.269 | 0.170 | 1.585 | 0.113  NS |
|  |  | Threats | Climate | 0.632 | 0.207 | 3.048 | 0.002  ** |
|  |  |  | Hunting or fishing | -0.573 | 0.192 | -2.987 | 0.003  ** |
|  |  |  | Habitat loss or degradation | -1.814 | 0.172 | -10.545 | <0.001  *** |
|  |  | Actions | Control invasive or problematic species or disease | 1.051 | 0.211 | 4.984 | <0.001  *** |
|  |  |  | Reintroduction or translocation | 0.988 | 0.293 | 3.377 | <0.001  *** |
|  |  |  | Legislation or trade control | 0.490 | 0.177 | 2.772 | 0.006  ** |
|  |  |  | Species management plan | 0.433 | 0.199 | 2.177 | 0.029  * |
|  | Mammals | Traits | log(generation length) | 0.751 | 0.205 | 3.672 | <0.001  *** |
|  |  |  | log(range) | -0.381 | 0.050 | -7.695 | <0.001  *** |
|  |  |  | IUCN Red List category (linear) | -2.086 | 0.368 | -5.673 | <0.001  *** |
|  |  |  | IUCN Red List category (quadratic) | -0.030 | 0.267 | -0.114 | 0.909  NS |
|  |  |  | IUCN Red List category (cubic) | 0.266 | 0.238 | 1.118 | 0.263  NS |
|  |  |  | Marine (vs terrestrial) | 2.602 | 0.874 | 2.976 | 0.003  ** |
|  |  |  | Terrestrial and marine (vs terrestrial only) | 1.632 | 0.699 | 2.334 | 0.020  * |
|  |  | Threats | Problematic or invasive species or diseases | 0.576 | 0.279 | 2.069 | 0.039  * |
|  |  |  | Habitat loss or degradation | -0.992 | 0.400 | -2.479 | 0.013  * |
|  |  | Actions | Species management plan | 1.024 | 0.352 | 2.911 | 0.004  ** |
|  |  |  | Reintroduction or translocation | 0.934 | 0.402 | 2.324 | 0.020  * |
|  |  |  | Awareness or education | 0.878 | 0.328 | 2.682 | 0.007  ** |
|  | Amphibians | Traits | log(range) | -0.516 | 0.042 | -12.237 | <0.001  *** |
|  |  |  | IUCN Red List category (linear) | -4.101 | 0.258 | -15.917 | <0.001  *** |
|  |  |  | IUCN Red List category (quadratic) | -0.028 | 0.177 | -0.157 | 0.875  NS |
|  |  |  | IUCN Red List category (cubic) | 0.137 | 0.157 | 0.874 | 0.382  NS |
|  |  | Threats | Climate | 0.745 | 0.181 | 4.106 | <0.001  *** |
|  |  |  | Pollution | -0.756 | 0.205 | -3.694 | <0.001  *** |
|  |  |  | Hunting or fishing | -0.745 | 0.345 | -2.157 | 0.031  * |
|  |  |  | Habitat loss or degradation | -1.812 | 0.280 | -6.476 | <0.001  *** |
|  |  | Actions | Reintroduction or translocation | 2.161 | 0.596 | 3.629 | <0.001  *** |
|  |  |  | Area management plan | 2.154 | 0.682 | 3.158 | 0.002  ** |
|  |  |  | Control invasive or problematic species or disease | 1.378 | 0.390 | 3.537 | <0.001  *** |
|  |  |  | In protected area | 0.540 | 0.225 | 2.399 | 0.016  * |
|  | Reptiles | Traits | log(range) | -0.346 | 0.032 | -10.685 | <0.001  *** |
|  |  |  | IUCN Red List category (linear) | -2.683 | 0.248 | -10.804 | <0.001  *** |
|  |  |  | IUCN Red List category (quadratic) | 0.474 | 0.203 | 2.341 | 0.019  * |
|  |  |  | IUCN Red List category (cubic) | 0.308 | 0.204 | 1.512 | 0.130  NS |
|  |  | Threats | Climate | 1.335 | 0.360 | 3.709 | <0.001  *** |
|  |  |  | Habitat loss or degradation | -1.122 | 0.214 | -5.237 | <0.001  *** |
|  |  |  | Problematic or invasive species or diseases | -1.186 | 0.252 | -4.706 | <0.001  *** |
|  |  |  | Pollution | -2.416 | 0.727 | -3.322 | <0.001  *** |
|  |  | Actions | Reintroduction or translocation | 2.756 | 0.451 | 6.113 | <0.001  *** |
|  |  |  | Control invasive or problematic species or disease | 1.334 | 0.425 | 3.139 | 0.002  ** |
|  |  |  | Awareness | 1.070 | 0.502 | 2.133 | 0.033  * |
|  |  |  | Species management plan | 0.975 | 0.464 | 2.101 | 0.036  * |
|  | Freshwater fish | Traits | log(range) | -0.320 | 0.039 | -8.282 | <0.001  *** |
|  |  |  | IUCN Red List category (linear) | -2.279 | 0.236 | -9.639 | <0.001  *** |
|  |  |  | IUCN Red List category (quadratic) | -0.370 | 0.195 | -1.895 | 0.058  NS |
|  |  |  | IUCN Red List category (cubic) | 0.661 | 0.165 | 4.014 | <0.001  *** |
|  |  | Threats | Pollution | -0.439 | 0.169 | -2.602 | 0.009  * |
|  |  |  | Habitat loss or degradation | -0.458 | 0.176 | -2.608 | 0.009  ** |
|  |  |  | Hunting or fishing | -1.316 | 0.227 | -5.804 | <0.001  *** |
|  |  | Actions | Species management plan | 1.250 | 0.285 | 4.387 | <0.001  *** |
|  |  |  | Reintroduction or translocation | 1.131 | 0.391 | 2.891 | 0.004  ** |
| Genuine change in IUCN Red List category | Amphibia, Aves and Mammalia | Traits | log(range) | 0.059 | 0.015 | 3.966 | < 0.001*** |
|  |  |  | IUCN Red List category (linear) | 3.043 | 0.812 | 3.750 | < 0.001*** |
|  |  |  | IUCN Red List category (quadratic) | 1.286 | 0.682 | 1.887 | 0.059 NS |
|  |  |  | IUCN Red List category (cubic) | 0.852 | 0.409 | 2.084 | 0.037* |
|  |  |  | IUCN Red List category (^4) | 0.316 | 0.169 | 1.877 | 0.060 NS |
|  |  |  | Marine (vs terrestrial) | 1.667 | 0.563 | 2.960 | 0.003** |
|  |  |  | Terrestrial and marine (vs terrestrial only) | 0.170 | 0.200 | 0.847 | 0.397 NS |
|  |  | Threats | Climate | -0.460 | 0.090 | -5.105 | < 0.001*** |
|  |  |  | Hunting or fishing | -0.457 | 0.098 | -4.678 | < 0.001*** |
|  |  |  | Invasive or problematic species or disease | -1.051 | 0.080 | -13.186 | < 0.001*** |
|  |  |  | Habitat loss or degradation | -0.934 | 0.151 | -6.187 | < 0.001*** |
|  |  | Actions | Reintroduction or translocation | 1.922 | 0.245 | 7.849 | < 0.001*** |
|  |  |  | In protected areas | 0.184 | 0.084 | 2.186 | 0.029* |
|  |  |  | Legislation or trade control | -0.225 | 0.108 | -2.080 | 0.038* |
|  |  |  | Awareness and education | -0.350 | 0.138 | -2.542 | 0.011* |
|  |  |  | Species management plan | -0.520 | 0.132 | -3.925 | < 0.001*** |
|  | Amphibia, Aves, Mammalia, only considering species traits | Traits | log(generation length) | -0.418 | 0.073 | -5.732 | <0.001  *** |
|  |  |  | log(range) | 0.033 | 0.015 | 2.147 | 0.032  * |
|  |  |  | IUCN Red List category (linear) | 5.295 | 0.709 | 7.466 | <0.001  *** |
|  |  |  | IUCN Red List category (quadratic) | 3.691 | 0.640 | 5.772 | <0.001  *** |
|  |  |  | IUCN Red List category (cubic) | 0.381 | 0.449 | 0.848 | 0.397  NS |
|  |  |  | IUCN Red List category (^4) | 0.092 | 0.258 | 0.356 | 0.722  NS |
|  |  |  | IUCN Red List category (^5) | -0.445 | 0.151 | -2.940 | 0.003  ** |
|  |  |  | Marine (vs terrestrial) | 2.307 | 0.556 | 4.147 | <0.001  *** |
|  |  |  | Terrestrial and marine (vs terrestrial only) | -0.145 | 0.158 | -0.917 | 0.359  NS |
|  | Amphibia | Traits | log(range) | 0.187 | 0.028 | 6.748 | <0.001  *** |
|  |  |  | IUCN Red List category (linear) | 1.411 | 0.163 | 8.654 | <0.001  *** |
|  |  |  | IUCN Red List category (quadratic) | -0.235 | 0.114 | -2.067 | 0.039* |
|  |  |  | IUCN Red List category (cubic) | -0.027 | 0.097 | -0.280 | 0.780 NS |
|  |  | Threats | Habitat loss or degradation | -1.416 | 0.281 | -5.041 | <0.001  *** |
|  |  |  | Climate | -0.639 | 0.119 | -5.383 | <0.001  *** |
|  |  |  | Problematic or invasive species or diseases | -1.692 | 0.115 | -14.657 | <0.001  *** |
|  |  | Actions | In protected area | 0.519 | 0.124 | 4.178 | <0.001  *** |
|  |  |  | Species management plan | -1.383 | 0.349 | -3.962 | <0.001  *** |
|  |  |  | Legislation or trade control | -0.624 | 0.269 | -2.325 | 0.020* |
|  | Aves | Traits | IUCN Red List category (linear) | 1.196 | 0.172 | 6.962 | <0.001  *** |
|  |  |  | IUCN Red List category (quadratic) | 0.644 | 0.152 | 4.247 | <0.001  *** |
|  |  |  | IUCN Red List category (cubic) | 0.353 | 0.138 | 2.562 | <0.010  * |
|  |  | Threats | Problematic or invasive species or diseases | -0.608 | 0.146 | -4.157 | <0.001  *** |
|  |  |  | Hunting or fishing | -0.381 | 0.137 | -2.786 | <0.005  ** |
|  |  |  | Habitat loss or degradation | -0.717 | 0.193 | -3.723 | <0.001  *** |
|  |  | Actions | Reintroduction or translocation | 2.390 | 0.321 | 7.446 | <0.001  *** |
|  |  |  | Species management plan | -0.666 | 0.159 | -4.203 | 0.000  *** |
|  | Mammalia | Traits | log(range) | 0.074 | 0.038 | 1.983 | 0.047  * |
|  |  |  | IUCN Red List category (linear) | 3.377 | 0.990 | 3.410 | <0.001  *** |
|  |  |  | IUCN Red List category (quadratic) | 1.068 | 0.808 | 1.322 | <0.186 NS |
|  |  |  | IUCN Red List category (cubic) | 0.680 | 0.515 | 1.321 | 0.187  NS |
|  |  |  | IUCN Red List category (^4) | 0.086 | 0.267 | 0.321 | 0.748 NS |
|  |  |  | Marine (vs terrestrial) | 2.571 | 0.719 | 3.576 | <0.001  *** |
|  |  |  | Terrestrial and marine (vs terrestrial only) | -1.630 | 0.459 | -3.551 | 0.002  ** |
|  |  | Threats | Habitat loss or degradation | -0.837 | 0.396 | -2.112 | 0.035* |
|  |  |  | Hunting or fishing | -1.063 | 0.213 | -4.991 | <0.001  *** |
|  |  | Actions | Reintroduction or translocation | 1.897 | 0.465 | 4.077 | <0.001  *** |
|  |  |  | Area management plan | -0.829 | 0.364 | -2.278 | 0.023  * |
|  |  |  | Awareness or education | -0.658 | 0.264 | -2.487 | <0.013  ** |
| Prevented declines in state in spatial units (Green Status of Species) | All animals with published Green Status assessments |  | (Intercept) | -3.589 | 0.729 | -4.921 | < 0.001*** |
|  |  | Actions | Species management plan | 2.435 | 0.793 | 3.069 | 0.002** |
|  |  |  | Reintroduction or translocation | 3.198 | 0.906 | 3.530 | <0.001*** |
|  |  |  | Area management plan | 2.069 | 0.455 | 4.547 | < 0.001*** |
|  |  |  | In protected area | 2.010 | 0.617 | 3.260 | 0.001** |
