# Supplementary material for: Past conservation efforts reveal which actions lead to positive outcomes for species
Source: PLoS Biol. 2025 Mar 18;23(3):e3003051. doi: 10.1371/journal.pbio.3003051 (PMC12135918; doi:10.1371/journal.pbio.3003051)
Supplement: S4 Table — (DOCX) [file pbio.3003051.s008.docx]

| Indicator | Selection parameter | Selection direction | Model | AICc |
| --- | --- | --- | --- | --- |
| Global population trend |  |  | null | 8394.319 |
|  |  |  | global model | 6210.491 |
|  | AICc | backwards | drop protected area | 6208.995 |
|  | AICc | backwards | also drop problematic or invasive species control | 6211.74 |
|  | AICc | backwards | also drop area management plan | 6211.674 |
|  | AICc | forwards | habitat loss or degradation + current Red List category + species management plan + invasive or problematic species control + reintroduction + climate change + ecosystem + pollution + awareness + hunting or fishing + legislation or trade control | 6211.74 |
|  | AICc | forwards | add protected area | 6212.98 |
|  | AICc | forwards | add area management | 6210.131 |
|  | AICc | forwards | add problematic or invasive species or diseases | 6210.583 |
|  | p | backwards | log(range) + current Red List category + ecosystem + problematic or invasive species control + reintroduction + species management plan + awareness + legislation or trade control + habitat loss or degradation + hunting or fishing + climate change + pollution | 6211.74 |
| Genuine change in Red List category |  |  | null | 6481.164 |
|  |  |  | global model | 6041.014 |
|  | AICc | backwards | drop area management plan | 6039.19 |
|  | AICc | backwards | drop pollution | 6040.829 |
|  | AICc | forwards | problematic or invasive species or diseases + initial Red List category + habitat loss or degradataion + reintroduction + climate change + hunting or fishing + log(range) + species management plan + awareness + ecosystem | 6043.327 |
|  | AICc | forwards | add protected area | 6041.429 |
|  | AICc | forwards | add invasive or problematic species control | 6042.685 |
|  | AICc | forwards | add legislation or trade control | 6041.866 |
|  | AICc | forwards | add pollution | 6043.192 |
|  | p | backwards | log(range) + initial Red List category + ecosystem + protected area + reintroduction + species management plan + awareness + legislation or trade control + habitat loss or degradation + hunting or fishing + problematic or invasive species or diseases + climate change | 6039.133 |
| Prevented decline in state in spatial units (Green Status of Species) |  |  | null | 448.4316 |
|  |  |  | global model | 312.0698 |
|  | AICc | backwards | drop hunting or fishing, log(generation length), legislation or trade control | 305.7144 |
|  | AICc | backwards | also drop log(range) | 304.9628 |
|  | AICc | backwards | also drop awareness | 304.6344 |
|  | AICc | backwards | also drop economic or livelihood incentives | 305.3249 |
|  | AICc | backwards | also drop problematic or invasive species or diseases | 305.2887 |
|  | AICc | backwards | also drop pollution | 303.7363 |
|  | AICc | backwards | also drop climate change | 303.8974 |
|  | AICc | forwards | reintroduction + protected area + area management plan + species management plan + counterfactual state | 301.515 |
|  | AICc | forwards | plus invasive or problematic species control | 299.8163 |
|  | AICc | forwards | plus economic or livelihood incentives | 299.6989 |
|  | AICc | forwards | plus awareness | 300.2193 |
|  | p | backwards | protected area + area management plan + species management plan + reintroduction | 308.3603 |
