# Supplementary material for: Past conservation efforts reveal which actions lead to positive outcomes for species
Source: PLoS Biol. 2025 Mar 18;23(3):e3003051. doi: 10.1371/journal.pbio.3003051 (PMC12135918; doi:10.1371/journal.pbio.3003051)
Supplement: S5 Table — Mammals had no significant differences between actions. (DOCX) [file pbio.3003051.s009.docx]

| **Action** | **Birds** | | |
| --- | --- | --- | --- |
|  | Residuals | p value | More/less likely |
| Reintroduced or translocated | 4.005 | 0.001 | >** |
| Area management plan | 4.005 | 0.001 | >** |
| In protected area | 1.649 | 1 | NS |
| Control of invasive or problematic species or diseases | 3.121 | 0.036 | > |
| Species management plan | 0.471 | 1 | NS |
| Captive breeding | -1.296 | 1 | NS |
| Awareness or education | -2.179 | 0.587 | NS |
| Harvest management plan | -2.768 | 0.113 | NS |
| Legislation or trade control | -3.062 | 0.044 | <* |
| Monitoring scheme | -3.946 | 0.002 | <** |
