# Supplementary figures and images for: Past conservation efforts reveal which actions lead to positive outcomes for species
Source: PLoS Biol. 2025 Mar 18;23(3):e3003051. doi: 10.1371/journal.pbio.3003051 (PMC12135918; doi:10.1371/journal.pbio.3003051)

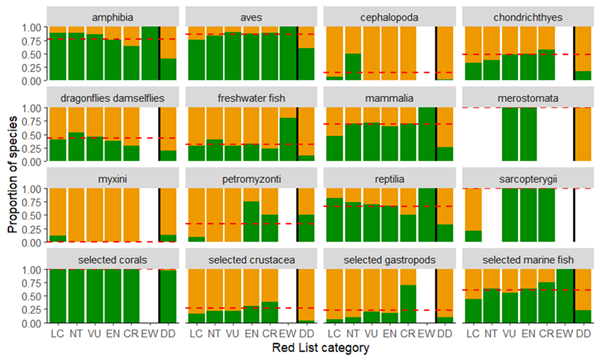

Supplement: S1 Fig — The dashed red line indicates the mean proportion across all species. See FigS1.csv for underlying data, available at: https://www.iucnredlist.org/resources/data-repository#Past%20conservation%20efforts. (TIF) [file pbio.3003051.s010.tif]

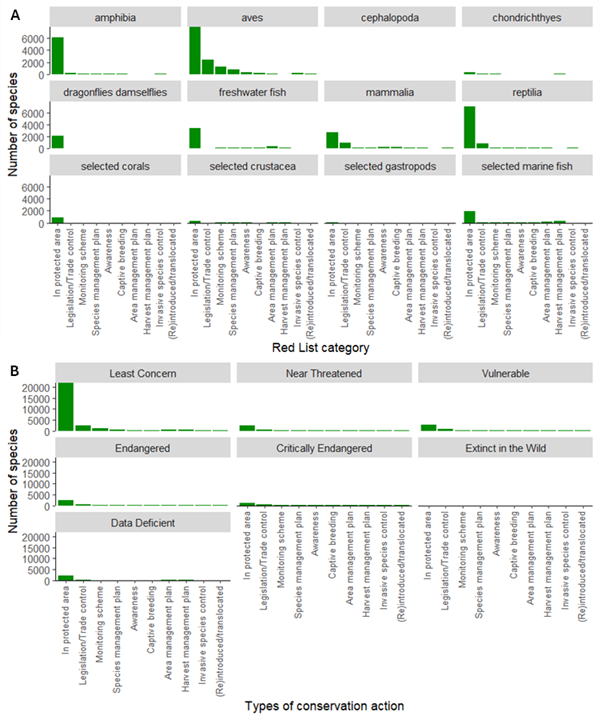

Supplement: S2 Fig — The percentage of species in animal groups which have been comprehensively assessed by the IUCN Red List that have each type of conservation action in place, (A) split by taxa that have been comprehensively assessed in the IUCN Red List (more than 80% assessed, excluding Cephalopoda, Merostomata, Myxini, Petromyzontid and Sarcopterygii) and (B) split by IUCN Red List category. See Fig S2A.csv and Fig S2B.csv respectively for underlying data, available at: https://www.iucnredlist.org/resources/data-repository#Past%20conservation%20efforts. (TIF) [file pbio.3003051.s011.tif]
