# Supplementary material for: Past conservation efforts reveal which actions lead to positive outcomes for species
Source: PLoS Biol. 2025 Mar 18;23(3):e3003051. doi: 10.1371/journal.pbio.3003051 (PMC12135918; doi:10.1371/journal.pbio.3003051)
Supplement: Attachment — Submitted filename: 2024.11.04_Reponse to reviewers.docx [file pbio.3003051.s012.docx]

**Response to Editor and Reviewer’s comments**

**Editor**

Comment:

You'll see that reviewer #1 is very positive, but has a number of concerns; most of these can be addressed textually, but his/her prime concern is your decision not to consider ex situ conservation measures. Reviewer #2 is also positive, and has no issues with the models and methodology, but thinks that the manuscript would benefit from shortening considerably to enhance its appeal (e.g. halve the length of the Results, and remove repetition from the Discussion). S/he also wants you to discuss the limitations of using the Red List for this analysis, and to emphasise the distinction between populations and species. A couple of the points might require minor extra analyses.

I discussed the reviews with the Academic Editor, who said "As the authors can see, both referees are enthusiastic about the manuscript, but recommend some revisions. Referee 2 especially brings up specific, but also general points about manuscript length, disclaimers about the IUCN dataset, and most importantly, the third point about populations versus species. Overall, I suggest the authors address referee comments and resubmit."

In light of the reviews, which you will find at the end of this email, we are pleased to offer you the opportunity to address the comments from the reviewers in a revision that we anticipate should not take you very long. We will then assess your revised manuscript and your response to the reviewers' comments with our Academic Editor aiming to avoid further rounds of peer-review, although might need to consult with the reviewers, depending on the nature of the revisions.

Response:

*We thank the Editor and Academic Editor for their feedback. See below how the reviewers’ comments were addressed.*

**Reviewer #1:**

Comment:
It was a pleasure to read and review this manuscript. This is the first time I have ever written that but it's true. The paper is exceptionally well written. The language is clear, transparent and unambiguous. The presentation of the analysis and results was straightforward and logical. My congratulations and appreciation to the authors.

Response:

*Thank-you for this review, we are glad you enjoyed the paper!*

Below are a few comments for consideration:

*       It is great that the authors are mining the IUCN Red List for useful information that can help us do more and better conservation. This is, of course, what the database is for. However, terms and definitions are important here, not least because they get very careful treatment in the Red List database, which is one of the things that makes it so valuable. In the abstract, the authors state, "This suggests a range of conservation interventions have successfully conserved some species at greatest risk but have rarely recovered populations to resilient levels." This seems contradictory. Is it saying you can successfully conserve species without recovering them to resilient levels?

Response

*We agree this was unclear. This is referring to successful actions that improved species’ status and reduced their extinction risk, but did not (yet) lead to full recovery to resilient levels. The text has been updated to clarify.*

*       Invasive Species Control seems like an outcome to me, not an action, as does "potential occurrence in protected areas". What does "potential occurrence" mean, as an action? From the manuscript's title, and the introduction, I was expecting an analysis of more specific actions that could (for example) give us new insights into how to achieve those outcomes. I think what this analysis is really telling us is that when we successfully mitigate threats and take positive action (like reintroduction), species bounce back, but this work is easier, and the bounce back is quicker, when we only have to do it over relatively small, bounded area for a specific population. This is interesting but not novel.

Response

*We analysed the information documented in IUCN Red List assessments, where conservation actions in place are documented using the IUCN Actions Classification Scheme, under which broad classes of action are used to represent a range of specific interventions. So, for example, trapping, shooting and application of poison bait may each be used to reduce or eradicate populations of invasive alien species, but are all coded as “2.2 Land/water management: Invasive/problematic species control”.*

*“Potential occurrence” in protected areas is scored if a species’ range overlaps with one or more protected areas according to a spatial (GIS) analysis: it is not necessarily confirmed that the species actually occurs within the protected area (although this is likely).*

*The reviewer’s summary is broadly correct but we think the analysis is novel because we believe it is the first analysis at this scale (in terms of number of species) to examine both the kinds of species that have improved in status owing to conservation and the types of actions that have driven these improvements.*

*       My main critique is that the authors erred when choosing to exclude ex situ conservation from their analysis: "Ex situ conservation (captive breeding) was excluded from the models as it does not have an impact on species' extinction risk until individuals are introduced into the wild (which is documented separately as 'species reintroduction' or 'benign introduction')" (my emphasis). It would be preferable if, wherever it says, 'extinction risk', it said instead, 'risk of extinction in the wild', or something similar. The Red List distinguishes between Extinct, and Extinct in the Wild, and the short cut is problematic as it suggests ex situ makes no difference to extinction risk. Some species cannot be reintroduced for decades, so it's not a trivial distinction.

o       The analysis showed that "Reintroductions or translocations were significantly more likely to have led to reductions in extinction risk compared with other actions",

o       We can probably assume that many reintroductions were from ex situ sources (though they didn't measure this),

o       So, a threatened species with a healthy ex situ population will have more reintroduction options than one without,

o       Therefore, that species must have less risk of extinction than if there were no ex situ population in place.
The wording they use probably just reflects RL terminology. Strictly speaking, the RL measures risk of becoming Extinct in the Wild, rather than the risk of extinction (usually one and the same thing, but not always). But the authors express it in a more generalized way: "does not have an impact on species' extinction risk".

Response

*Thanks for highlighting this. You are correct that where we refer to extinction and extinction risk, this relates to extinction in the wild. We have adjusted the text in the methods where we mention exclusion of captive breeding from the models to make this clearer.*

*       This paper makes clear the need for the Red List Conservation Actions work currently underway to result in a practical, comprehensible set of standards.

*       Given the fact that a shockingly low 51.8% of species in comprehensively assessed animal groups have conservation actions in place recorded, I would like to see the authors call for a change in instructions to RL assessors making recording of conservation actions in place required rather than recommended.

Response

*We agree that this is a shortcoming of current Red List assessments. It reflects the challenge of balancing the need to expand the number of species assessed on the Red List and to regularly reassess all of those that have been assessed with the need to make the exercise of undertaking a Red List assessment not too onerous for volunteers attempting to assess hundreds or thousands of species. We have updated the text to make a more direct call for strengthening the documentation of actions in place.*


**Reviewer #2:**

Comment

I have reviewed the manuscript "What works to improve species conservation state? An analysis of species whose state has improved and the actions responsible". Using IUCN Red List data for almost 70,000 species, the manuscript aims to link the conservation status of species with the conservation actions implemented and threats affecting those species. Overall, this is a very interesting and relevant study that provides really important insights into how our conservation responses relate to the state of biodiversity. Towards this, the manuscript provides a very large-scale perspective. For obvious reasons that approach cannot explore important details at the local level but potentially provides some overarching observation that are useful for local scale approaches. I think the topic and quality of the analysis makes this paper very fit for PLOS Biology, and I overall want to commend the authors on a great study.

The methodology seems appropriate and I have no concerns about how the study has been conducted - which is probably also because the most interesting of the results are largely descriptive and hinges on how the Red List data is categorized (which follows a mixed of using established categories and logical decisions to group within those) and grouped. The models also seem appropriate given the data.

Response

*We thank reviewer 2 for their comments and their kind words.*

However, I have three overall concerns as well as some minor comment from reading the manuscript. None of the overall comments I consider to be particularly major - though still important to consider and ideally address.

Comment

First, the manuscript is very long. The Discussion for example is just over 10 pages and the Result section (without figures) is ca. seven pages. The Introduction seems appropriate and is a good setup for the paper, but I would suggest that Methods, Results, and Discussion could be significantly reduced (maybe even by half) to provide a more focused and clearer narrative. This may also require removing some element, but if it helps provide a clearer story it might be worth it. I will leave the comment on length at that. I do not believe it is my role as a reviewer to decide the focus on someone else's paper, that should be the prerogative of the authors, but I do believe the impact and uptake of this paper will be severely reduced if the main story cannot be presented in a shorted and more precise manor. And given that I really like the work that has been done, I would think that a shame. Related to this - while I agree that a key role of the Discussion is to highlight the
main results of the paper and put them in context of existing knowledge, I did find the Discussion a bit heavy on repeating things already presented in the Results section. I would suggest this is reduced and that the Discussion is reserved to discuss the most interesting results not necessarily all results and to bring them out in a way that highlights their importance rather than repeating numbers already presented in the previous section.

Response

*The length of the manuscript reflects the fact that it was originally submitted as a full length article but we were advised to change the format to a short report. However we accept the reviewer’s advice and have cut large amounts of text from the methods, results and discussion, moving some to supplementary material, and have focussed the discussion on the more interesting results, as suggested.*

My two other comments related to things I believe needs to be explained and discussed a bit more - and yes I realize that asking in one comment to cut and in the next to add might not make the task of the authors any easier.

Comment

Second, I believe the Red List is an appropriate data-source for this analysis, and importantly the only data source that can be used to answer these questions at this scale. However, that does not mean it is perfect. I think the Discussion needs to include a paragraph of self-reflection on the weaknesses of the Red List for this analysis and the implications of this for the interpretation of the study.

Response

*We did caveat interpretation of Red List results throughout the discussion, but agree we did not acknowledge its specific limitations for this analysis. We have added recognition of this in the reworked discussion under the section on final considerations .*

Comment

Third, except for maybe the most threatened (and small ranging species) conservation actions are implemented at the level of populations not species. Thus, the paper is making a leap in connecting species and actions. Given the data, this leap is probably unavoidable, but it would be good to reflect on this in the Discussion. There is maybe a bit of this in the part specifically about protected areas and how an overlap in range might not equate to an overlap in focus, but this is a more overall issue for the manuscript.

Response

*This is a fair point, although in the cases where action led to a species being reclassified in a lower category of risk, the impacts on a population are judged to have had substantial impacts that are significant at the level of the species. Nevertheless, we have added a reflection on this in the discussion where we discuss why action presence is associated with no change or worsening in status.*

Minor comment:

Line 42: technically, its not the world but the signatories or nations of the Convention on Biological Diversity that committed to this - I realize there is a big overlap, but still using CBD language here would be more appropriate.

*Edited as requested.*

Line 78: "IUCN" missing before "Red List" - check throughout the manuscript.

*We realised this wasn’t consistent throughout so thanks for highlighting this. While we could do this, it would add >100 words to the text, so for brevity we explain when first mentioned that we use “Red List” to refer to the “IUCN Red List of Threatened Species”. If the Editor is happy for us to increase the word count, we are happy to include “IUCN” each time.*

Line 78-82: the main message of this sentence is not entirely clear to me. The Red List should (hopefully) document which species have undergone *genuine* changes in their extinction risk without linking it to actions. I am sure there is something interesting here to include, but in its current form, this sentence seems to mix things without clearly articulating their connection.

Response

*Edited to improve clarity*

Line 86: check presentation of IUCN reference (and in text-references in a few places seems to have different formats - for example sometimes including the initials of first and middle names).

Response

*Edited throughout – check ref formatting*

Lines 88-90: not entirely clear to me. Is this a general criticism of the Red List? In that case a bit more nuance might be needed.

Response

*It is acknowledging the basis of this information may be subjective and is not objectively determined, thought this has been moved to the discussion as fitted better in limitations of Red List data section.*

Lines 112-116: should numbers be given with an "n" - i.e. "(n = 7,983)"?

*We have edited the text to make this clearer by moving the number out of brackets.*

Line 125: rewrite - maybe: "For just under half of the mammals, the IUCN Red List did not contain information about generation length, so …."

*The wording was condensed as part of our efforts to shorten the manuscript as requested.*

Line 129: I would suggest using parenthesis around scientific name for the Greenland shark. Also relevant other places in the manuscript.

*We have deleted this particular instance, but have followed this recommendation elsewhere.*

Lines 126-129: could probably be deleted - you have described above that you checked the data with the IUCN Red List unit, I guess this is just one particular case of what would be reasons for checking.

*Deleted as suggested*

Lines 134-135: which equal area projection?

*Cylindrical – this is how it is typically referred to.*

Lines 137-140: this assumption is not without its limitation. I do not suggest changing anything analytically, but maybe including an acknowledgement, in the Discussion section, that there is a leap here.

*We have now acknowledged this assumption when discussing the range size model relationships in the discussion.*

Lines 290-292: I realize that different parameters have been presented in other parts of the methods in sections detailing how they were developed. But it would be useful to recap here what is the total list of variables included in the full model.

*Edited as suggested*

Lines 292-294: I am not a huge fan of conducting only backwards model selection. I would suggest at the very least that both backwards and forward model selection was performed to ensure that they reach the same model, or even better that a method like dredging or Lasso is used (Lasso might be most appropriate especially if the authors are concerned about the "fishing expedition" risk of dredging.

*We tried to dredge the models but this was unfeasible given the model contained 14-16 variables (and so 2^14^-2^16^ models). We also ran into similar issues with lasso regression. Therefore we ran forwards and backwards selection on AICc ( forward p-value selection was not possible as several variables all had a p-value <2e^-16^ so could not be prioritised). Both forward and backwards AICc selection came to similar final models (give or take one or two variables), and similar to p-selection except retaining more variables due to the nature of AICc. Only the Green Status p-selected model had an AICc > 2 than the best forward/backward AICc model, but given similar or better values of AICc for the population trend and Red List category model, and one variable difference to the best Green Status model, we reported the p output as the most parsimonious model output, for consistency across all models, and as we were interested in relative contribution of each factor to species status (not just which factors where important in the best model).*

Lines 344-346: I was a bit confused by the introduction of "other invertebrates" here as the first part of the sentence reef-building corals - which I guess technically are invertebrates but which are rarely referred to as such. Maybe drop the "other"?

*Edited as suggested.*

Lines 485-287: this paper present some really interesting results. The first sentence of the discussion should really re-state what the main take-home message is. However, by relating the finding to how they are only marginally different to another study (i.e. Senior et al. 2024) that effect is lost. I would strongly suggest rephrasing to relate to the findings of this study in their own right.

*We have deleted reference to the paper so the results stands out more clearly*

Line 500: each species had more actions or across the species there were more actions? This needs to be clarified.

*The former, clarified in text.*

Line 517: delete the first "groups"

*Deleted as suggested.*

Lines 526-528: good point and one I would relate to the over-representation of LC species in protected areas, as this is likely to be more the case for LC species and threatened species and thus, the importance of PAs is likely more inflated for that group.

*Added this as suggested to this section.*

Line 529: these numbers need to be updated. Especially the marine coverage statistics is wildly outdated.

*Thanks for spotting, updated with numbers from 2024 Protected Planet report.*

Figure 1: check the numbers close to the x-axis and fix them where they are not fully visible.

*We have adjusted this to make the values more visible, though given how the plot was generated, this was challenging but we feel all values are visible.*

Figure 2: I think these are interesting and probably good as main figures, but richness map of any sub-division below all species is always very likely to be strongly correlated to species richness. I can see strong deviations here from that pattern, but I would suggest presenting in the supplementary material a map of the residuals between all species richness and changes in Red list status to show highlight where chose changes really differ from the predictions based on SR.

*We agree this comparison is interesting, but as species richness maps for vertebrates are well established, rather then recreate these we felt it was more appropriate to cite these and then make the comparison in the text to highlight areas where greater numbers of species are improving/deteriorating relative to areas where high species richness is found.*

Figure 3: I really like the idea of this figure, but also have to admit I didn't find it super easy to decode. Starting with the three categories on top that are not super easy to place in relations to the columns below. I also hadn't from the method seen that Red List category was included in five different versions. What is the rationale for this? what theory supports trying red list category to the power of 5? Same with Initial Red List category? This seems overly complicated.

*We have combined the current and initial Red List category fields into one row, with notes to recognise these differ, and dropped all non-significant variables from the plot, including ordinal powers of Red List category (though full stats are presented in the supplementary materials as before).*

*The Red List category has multiple variables as is an ordered factor, reflecting the ordinal nature of the categories (ranging from Least Concern to Extinct), so the model has to consider varying numbers of different relationships depending upon the number of categories included in the model (and all must be retained in the model). So unfortunately there is no way to suitably simplify this, however for the visual plot in the main text I have dropped where consistently non-significant (with full model output in the supplementary material). Also, the text focuses on the linear relationship between Red List category and the conservation status indicator as when used, the category has stronger relationships with each conservation indicator.*
