# Supplementary material for: Past conservation efforts reveal which actions lead to positive outcomes for species
Source: PLoS Biol. 2025 Mar 18;23(3):e3003051. doi: 10.1371/journal.pbio.3003051 (PMC12135918; doi:10.1371/journal.pbio.3003051)
Supplement: Attachment — Submitted filename: 2025.01.20_Reponse to reviewers.docx [file pbio.3003051.s013.docx]

**Response to Reviewer 2’s comments**

**Reviewer #2:**

I overall think the authors have done a good job of responding to the reviewers' comments and I think the manuscript is improved because of this. I don't have any major issues with the updated manuscript and I think the quality and clarity as well as the scope now make this a great paper for publication in PLOS Biology. And I again want to congratulate the authors on this work and I am looking forward to seeing it out, and I would strongly recommend this work be published in PLOS Biology.

Response:

*We again thank Reviewer 2 for their positive feedback and constructive comments that have greatly improved the manuscript. See how we address their remaining comments below.*

My only overarching comment is very similar to my comment on the original manuscript and relate to length. Even though I can see the authors have done a lot to address my previous comment on this (in particular in relation to the Result section), the manuscript still feels quite long with the Discussion section still close to 10 pages. I will leave this between the editor and authors. To me the discussion still, in places a bit too repetitive of the results. As a consequence, the reader is not helped very well in terms of clearly identifying and navigating what the authors see as the main and most important take-home messages. But I can also see that there is many interesting points being made, so if the authors feel this is the right format for their paper, then I will not argue with that - even if I would likely have preferred a version that was still significantly shortened compared to even the new version.

*It is great to hear the shortening of the manuscript has improved this. We agree the Discussion was still too long and so have cut this further to around 7 pages, limiting repetition of results.*

A few minor observation when reading it:

Line 157: helena in " Troides Helena" should not be capitalized.

*We have changed to lowercase as suggested, thanks for spotting this.*

Lines 269-275: would it make more sense to just include references to software in the sections where their use is described rather than having six lines dedicated to this.

*Given many of these packages are used throughout the analyses and for clarity and flow of the writing, we felt it flowed better and is more efficient and succinct to include in one section.*

Line 303: are the numbers within parenthesis the number of species? Then I would include "n=" so it reads: (n = 1,220) - for example.

*Yes, added species after the number in the parentheses to clarify.*

Lines 321-328: this seems very interesting but also currently quite confusing. For example, how are these two statements "contrasting"?: 1) "Species with smaller global ranges, those at lower risk of extinction and those found in both terrestrial and marine ecosystems were more likely to have increasing global populations." And 2) "species with larger global ranges, those previously at higher risk of extinction and marine species were more likely to have improved in Red List category". Both seems to be encouraging even if the specific metric of improvement is different (i.e. population increases vs. improved red list category).

*We considered these to be contrasting given one appears those with small ranges and lower threat status are in better state where the other indicates those with larger ranges and higher threat have improved. But agree as the indicator is different these tell us different things rather than are contrasting so have dropped the word “contrasting”.*

Lines 374-378: can this sentence be broken up into multiple?

*We have tried to shorten the sentence and split it into two.*

Line 380: I think "are" should be deleted? Or "with" replaced with "where"?

*Dropped “are” as suggested.*

Lines 434-444: still seems quite repetitive of the result section. Is there no way the parts that repeats the results could be even shorter?

*We have condensed the text in this section and tried to simplify text so less repetitive of the results.*

Line 493: how does a new section start with "Similarly"?

*We have dropped “Similarly” from the text.*
